# From bedside to battlefield: intersection of ketone body mechanisms in geroscience with military resilience

**DOI:** 10.1007/s11357-020-00277-y

**Published:** 2020-10-02

**Authors:** Brianna J. Stubbs, Andrew P. Koutnik, Jeff S. Volek, John C. Newman

**Affiliations:** 1 Buck Institute for Research on Aging, Novato, CA USA; 2 Institute for Human and Machine Cognition, Pensacola, FL USA; 3 Department of Molecular Pharmacology and Physiology, USF, Tampa, FL USA; 4 Department of Human Sciences, Ohio State University, Columbus, OH USA; 5 Division of Geriatrics, UCSF, San Francisco, CA USA

**Keywords:** Metabolism, Ketone bodies, Aging, Geroscience, TBI, Sarcopenia

## Abstract

Ketone bodies are endogenous metabolites that are linked to multiple mechanisms of aging and resilience. They are produced by the body when glucose availability is low, including during fasting and dietary carbohydrate restriction, but also can be consumed as exogenous ketone compounds. Along with supplying energy to peripheral tissues such as brain, heart, and skeletal muscle, they increasingly are understood to have drug-like protein binding activities that regulate inflammation, epigenetics, and other cellular processes. While these energy and signaling mechanisms of ketone bodies are currently being studied in a variety of aging-related diseases such as Alzheimer’s disease and type 2 diabetes mellitus, they may also be relevant to military service members undergoing stressors that mimic or accelerate aging pathways, particularly traumatic brain injury and muscle rehabilitation and recovery. Here we summarize the biology of ketone bodies relevant to resilience and rehabilitation, strategies for translational use of ketone bodies, and current clinical investigations in this area.

## Endogenous ketone production and metabolism

Ketone bodies are endogenous metabolites that have been implicated in modulation of multiple pathways relevant to aging and aging-related resilience [1]. A growing catalog of direct molecular actions support their roles in geroprotective pathways, as does circumstantial evidence from their induction in aging-relevant physiological settings such as fasting, dietary restriction, and exercise [2–4]. The increased physical and emotional stress experienced by military service members during their career may act to accelerate the aging process in these individuals [5, 6]. Furthermore, the military population has an increased risk of sustaining injuries and accumulating complex co-morbidities that could go on to contribute to physical disability or cognitive decline [7–10]. Therefore, there may be significant areas of application for geroscience interventions to promote resilience, improve rehabilitation, and prevent accelerated aging in active duty military and veterans alike.

Ketone bodies are small molecules synthesized primarily in the liver from fats during fasting, caloric restriction, prolonged exercise, or other circumstances when carbohydrates are scarce [2]. They circulate through the bloodstream, are transported across the blood-brain barrier, and are taken up by peripheral tissues in need of energy. They are oxidized in mitochondria to generate acetyl-CoA and thus drive the regeneration of ATP [11]. The two primary endogenous ketone bodies are acetoacetate (AcAc) and beta-hydroxybutyrate (BHB), which are interconverted in the final step in ketogenesis in the liver and the first step in ketolysis in peripheral tissues. Liver ketogenesis naturally occurs at a low level even after consumption of carbohydrate-rich meals, with AcAc and BHB found in the blood in roughly similar levels (~ 50–100 micromolar each) [12]. When ketogenesis is activated, overall ketone body levels increase into the millimolar range with a preferential increase in BHB [13]. Some of AcAc will spontaneously degrade into acetone, which is considered the third ketone body and provides the “fruity” breath scent common in diabetic ketoacidosis.

Ketone bodies have a uniquely wide physiological range that spans 3 orders of magnitude in blood concentration from as low as 0.01 mM in the carbohydrate-fed state to greater than 10 mM in diabetic ketoacidosis. Ketoacidosis is a life-threatening condition where metabolic control mechanisms such as insulin are absent or dysfunctional, and bicarbonate buffering capacity is outpaced by acid production [14]. Physiological or nutritional ketosis, such as occurs in fasting or on a ketogenic diet, lies in between these extremes of ketone levels. Physiological ketosis is an evolutionary-conserved state enabling inter-organ fuel requirements to be met without exogenous carbohydrate. In individuals with sufficient insulin, nutritional ketosis is maintained without risk of ketoacidosis due to carefully regulated feedback control mechanisms and gradual adaptation [15].

## Experimental and translational tools for ketone bodies

Ketone bodies for clinical or experimental purposes can be produced endogenously through a ketogenic diet or administered exogenously. The ketogenic diet involves limiting carbohydrate consumption, maintaining moderate protein intake, and meeting most of the body’s energy requirements with fat; this stimulates endogenous ketone production. Such diets have been used to treat intractable epilepsy and diabetes for over 100 years, and more recently have been investigated as treatments for type 2 diabetes and as co-therapies to treat various cancers [16–18]. A well-formulated ketogenic diet maintains blood BHB levels ~ 0.5–3 mM. However, the adoption of the ketogenic diet in both aging and military populations is not straightforward, not least because it necessitates dramatic changes to the habitual Western diet. Concerns for long-term use include possibly increasing the risk of cardiovascular disease in an at-risk aging population, or that ketoadaptation might impair physical performance [19, 20]. Although the impact of ketogenic diets on cardiovascular risk is controversial, ketogenic diets can beneficially modulate several risk factors for cardiovascular disease [21, 22]. Ketogenic diets have also been shown to be sustainable in elite athletes [23] and in military cadets [24] and produce changes in fat oxidation and body composition that may benefit performance.

An alternative approach to deliver the energetic and signaling effects of ketone bodies themselves, without the need for dietary modification, involves a class of compounds called “exogenous ketones” [25]. Exogenous ketone is a term widely used to describe the administration of ketone bodies or ketone body precursors. In reality, some forms of exogenous ketones leverage endogenous ketogenic pathways as well, but in a manner that avoids the usual suppression of ketogenesis by carbohydrate intake. During prolonged fasting or on a ketogenic diet, AcAc and BHB are produced endogenously in prodigious quantities—approximately 50 to 150 g/day [26], representing ~ 200–600 kcal of energy content. Thus, the key considerations for designing therapeutic exogenous ketones are to accommodate the food-like quantities that must be delivered, and to cope with the equimolar salt or acid load involved [25]. Existing categories of exogenous ketones include ketone salts, medium chain triglycerides, and ketone esters. An increasing number of exogenous ketone compounds are available and can be used alone or as an adjuvant to dietary methods; ketogenic diets prescribed for childhood epilepsies, for example, commonly include medium chain triglycerides to allow relatively more flexible carbohydrate intake [27].

Ketone salts are the salt form of beta-hydroxybutyrate, either as a sodium salt or with a mixture of cations. They are inexpensive to synthesize, are widely available to the public in consumer health products, and modestly increase blood BHB levels (~ 1 mM BHB increase) [25, 28]. Medium-chain fatty acids (unlike long-chain fatty acids) are not endogenously stored in appreciable amounts and their mitochondrial uptake and oxidation not inhibited by insulin. Medium-chain triglycerides (MCTs) are therefore a preferentially oxidized and ketogenic form of dietary fat that can provide a longer-lasting modest ketosis (~ 0.5–1 mM BHB increase) without added salt load [29]. MCTs have a body of clinical research supporting safety and efficacy for several metabolic outcomes in obese and diabetic patients, and early stage efficacy for improving metabolism in the aging brain and in Alzheimer’s disease [30–33]. Medium chain triglycerides are also widely commercially available, though acute dosing can be limited by gastrointestinal side effects [34] which appear to reduce with chronic administration [34–37]. Esters containing ketones and ketone precursors are also commercially available and result in higher blood BHB levels (~ 1–5 mM BHB increase) [28]. As there are several ketone bodies or ketone precursors, and the possibility of joining them in mono-, di-, or even tri-ester forms, the “ketone ester” family includes many possible compounds. Most recent clinical KE research has used a mono-ester of beta hydroxybutyrate and (R)-1,3 butanediol; however, other compounds are under development including a di-ester of acetoacetate and (R,S)-1,3 butanediol and a di-ester of hexanoic acid (a ketogenic medium chain fatty acid) and (R)-1,3 butanediol. The existing clinical literature on applications of ketone bodies includes studies using ketogenic diets and all of these varieties of exogenous ketones. Studies are underway of exogenous ketones in both diseases of aging and in military service members subjected to extreme environments.

## Ketone body mechanisms relevant to resilience and rehabilitation

Emerging evidence shows that the ketone bodies BHB and AcAc are not only passive carriers of energy but also possess a variety of drug-like signaling functions that are hypothesized to directly and indirectly affect processes that become gradually dysregulated during aging or rapidly dysregulated as a result of trauma or stress in military service members. These include oxidative stress, inflammation, gene expression, lipid metabolism, and neuronal function [2]. These signaling activities are mediated by both covalent and non-covalent protein interactions. For example, BHB inhibits class I histone deacetylase (HDACs) enzymes, thereby regulating promoter histone acetylation and gene expression [38]. Beta-hydroxybutyrylation (BHBylation) is a novel covalent modification of the lysine tails of histone proteins, akin to acetylation, and its abundance on histones increases in response to exogenous BHB or in fasted mice [39]. These epigenetic effects result in up-regulation of the stress-response genes Nrf2, Foxo3, and Mt2, resulting in cytoprotection from oxidative stress in several model systems [40–45]. BHB directly interacts with the RNA binding protein hnRNPA1, thereby enhancing stabilization of the Yamanaka factor Oct4 mRNA, and leading to reduced senescence in mouse vascular endothelial cells [46]. BHB also binds to at least two cell-surface receptors, HCAR2 and FFAR3 (reviewed in [2]). HCAR2 activation on microglia is implicated in neuroprotection by ketogenic diet in a mouse stroke model; FFAR3 also has a role in dampening inflammatory activation in lung and bowel disease, and perhaps in other contexts as well [2].

AcAc possesses several signaling activities that are distinct from BHB. These include binding to GPR43 to modulate lipid metabolism [47] and activation of the MEK1-ERK1/2 cyclin D1 pathway to accelerate muscle cell proliferation and muscle regeneration [48]. AcAc also prominently inhibits the neuronal vesicular glutamate transporter VGLUT2, thereby inhibiting glutamate release from neurons [49]. Some protein binding activities are shared between AcAc and BHB, but at much different potency. For example, BHB is an ~ 10-fold more potent HDAC inhibitor than AcAc [38], while AcAc is a 10-fold more potent VGLUT2 inhibitor [49].

For some important signaling activities the specific protein interactions of BHB have not yet been fully defined. Perhaps most relevant to a resilience and rehabilitation context, BHB inhibits assembly and activation of the NLRP3 inflammasome through a yet unknown non-energy-dependent mechanism [50] in rodent models of inflammatory disease and in isolated human leukocytes [51], although clinical evidence remains mixed [52]. The NLRP3 inflammasome is a key mediator of innate immune inflammatory activation [53], both acutely and in chronic inflammation such as in gout [51] and T2DM [54]. Decreased activation of the NLRP3 inflammasome could partially explain the reduction in inflammatory markers in response to ketogenic diets in individuals with metabolic syndrome after 12 weeks [55].

The oxidation of ketone bodies generates changes in cellular metabolic biochemistry that can have fundamental indirect signaling effects, through altering the abundance of metabolites like acetyl-CoA and succinyl-CoA, and redistributing cellular NAD^+^/NADH and NADPi^+^/NADPH pools (reviewed in Newman and Verdin [2]). These effects will be context- and tissue-specific. AcAc and BHB are interconverted by an NAD-dependent enzyme (Bdh1), driven both by substrate concentration and by the mitochondrial NAD^+^/NADH ratio. Reduction of AcAc to BHB increases the NAD^+^/NADH ratio, promoting activation of other NAD-dependent enzymes with geroprotective properties such as the sirtuin deacylases [56, 57]. However, the resulting lower concentration of NADH impairs mitochondrial energy production [58]. Reduction of AcAc occurs in the liver during ketogenesis on a ketogenic diet, as well as potentially in peripheral tissues such as skeletal muscle that uptake AcAc and re-release as BHB [59]. On the other hand, oxidation of BHB to AcAc in peripheral tissues generates NADH, which is available for and increases the efficiency of ATP production but reduces the NAD^+^/NADH ratio. Importantly, in conditions of low intracellular glucose where the activity of the hexose monophosphate is compromised, ketones can provide an alternative route to maintain cytosolic reducing potential [58, 60] as set by the ratio of NADP^+^/NADPH. The metabolism of BHB in heart or brain leads to the formation of mitochondrial acetyl CoA and citrate [60, 61]. Mitochondrial citrate is transported to cytoplasm by the citrate–isocitrate carrier where its conversion to alpha-ketoglutarate leads to the regeneration of NADPH by the NADP-linked isocitrate dehydrogenase. Maintenance of cytosolic NADP/NADPH couple is an important mechanism by which ketones may be directly protective against cellular damage from acute and chronic oxidative stress (reviewed in [52, 58]).

Finally, ketone bodies and ketogenic diet can signal through multiple interactions with the microbiome that affect systemic function. BHB directly reduces the abundance of certain bacteria taxa including pro-inflammatory bifidiobacteria along with reducing intestinal pro-inflammatory Th17 cells in mice [62]. Reduced bifidiobacteria was also observed in a clinical study of modified Mediterranean ketogenic diet in older adults with or without cognitive impairment [63]; changes in the gut fungal mycobiome were also observed [64]. Changes in the gut microbiome on ketogenic diets also affect the levels of microbial-associated gamma-glutamylated metabolites that enter systemic circulation, resulting in an elevated brain GABA/glutamate ratio and suppression of epilepsy in mouse models [65].

Signaling and energetic properties of ketone bodies can integrate to generate higher-level changes in physiological function, most of which are only beginning to be mechanistically mapped. Although many of these effects are systemic, based on the hepatic production and systemic circulation of ketone bodies, new evidence is suggesting local physiological effects too, driven in some instances by extrahepatic ketogenesis. For example, hepatic production of AcAc is consumed locally by liver innate immune cells (Kuppfer cells), resulting in downstream metabolism of AcAc into the glycosaminoglycan pathway which regulates tissue fibrosis. Exogenous AcAc, but not BHB (Kuppfer cells lack expression of Bdh1), is protective against diet-induced liver fibrosis [66]. Overexpression of Bdh1 in the heart increases ketone body oxidation and similarly reduces fibrotic remodeling in a mouse heart failure model [67]. Interestingly, BHB may have the opposite effect on Kuppfer cell fibrotic phenotypes [66], demonstrating the importance of matching specific ketone bodies and activities, in this case, oxidation of AcAc, to specific clinical applications. Ketone body oxidation in lymphatic endothelial cells increased lymphangiogenesis, improves lymph vessel function, and reduces edema in mouse models of lymphedema [68]. A recent elegant study showed that local production of ketone bodies in Lgr5^+^ intestinal stem cells mediated their continued stemness via BHB inhibition of HDACs to reinforce Notch signaling [69]. This not only demonstrated a potentially general role for BHB in regulating stem cell function but also showed the relevance of local BHB production to clinically relevant phenotypes. Perhaps the clearest set of mechanistic studies of ketones in a specific disease state has shown that oxidation of BHB in cardiomyocytes to generate energy is an adaptive mechanism that supports cardiac function in preclinical heart failure models [70–72] and in small human physiology studies in healthy individuals and in heart failure patients [70, 73]. Acute intravenous infusion of BHB provides dose dependent (0.7–3.4 mM) hemodynamic and cardiac output improvement in both healthy humans and heart failure patients with reduced ejection fraction [74, 75]. The heart may be a site of local ketogenesis as well, as a component of this adaptation [76]. Similarly, brain uptake and oxidation of ketone bodies may mitigate the partial energy deficit that occurs in the aging or injured brain as a result of reduced glucose uptake and utilization; this defect is more pronounced in diseases of aging such as Alzheimer’s disease [30, 31], as well as traumatic brain injury [77].

**Fig. 1:**
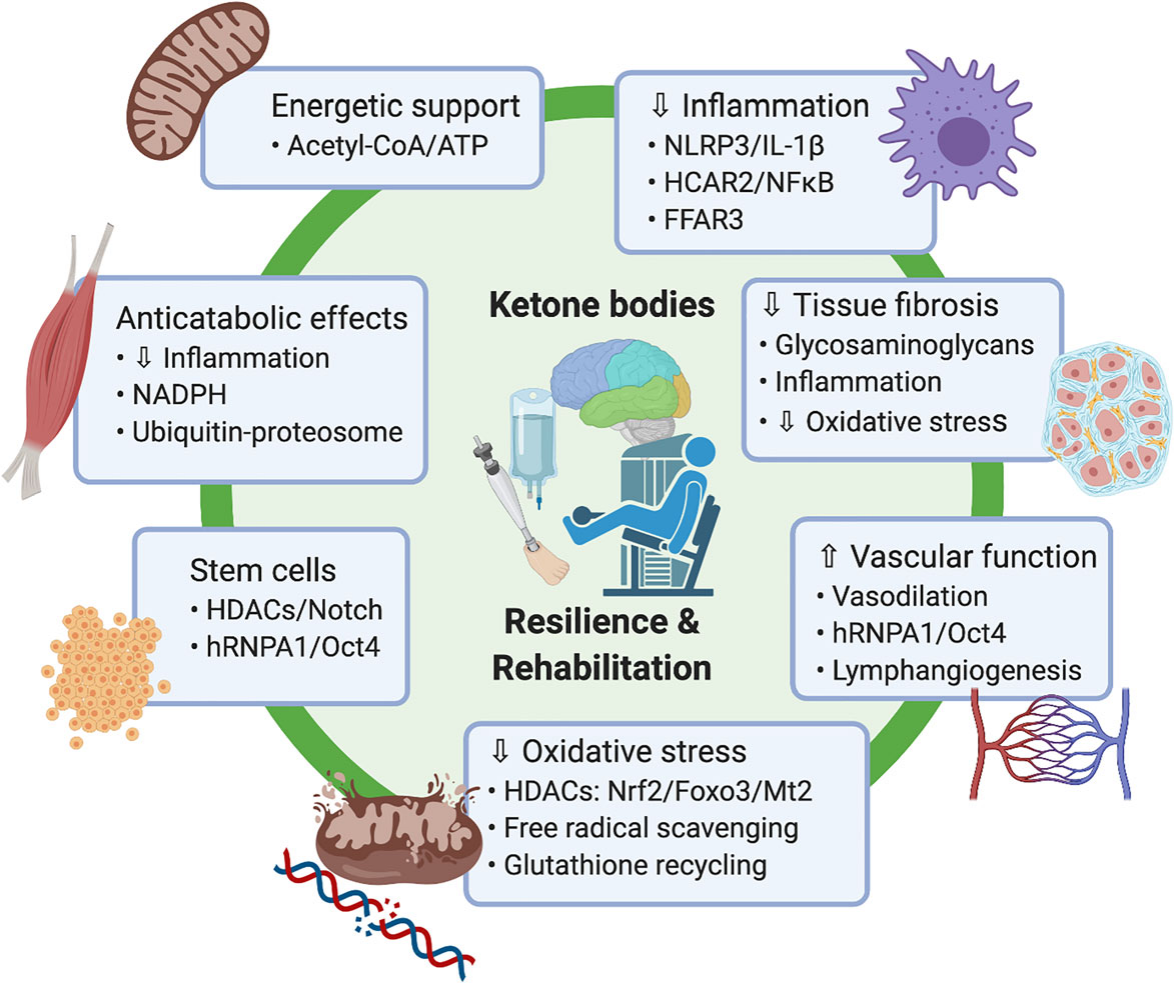
Mechanisms of ketone bodies relevant to resilience and rehabilitation

Altogether, these biological activities of ketone bodies provide a number of mechanisms that may be relevant to resilience and rehabilitation contexts from bedside to battlefield. Ketone bodies promote stem cell function, promote blood and lymph vessel function, moderate acute and chronic inflammation, reduce tissue fibrosis, protect against oxidative and hypoxic stress, and provide resiliency against hypoglycemia and energetic stress (Fig. 1). These molecular mechanisms of ketone bodies have only been linked to specific diseases in a few cases such as heart failure, Alzheimer’s disease, and gout, and even then, largely through preclinical models. Clinical trials have yet to definitively demonstrate benefits of endogenous or exogenous ketosis in human diseases of aging or following military relevant acute stressors. A key research need is to provide clear mechanistic links in the most promising organ- and tissue-specific contexts to inform specific hypotheses and clinical study designs in promising applications such as traumatic brain injury or muscle rehabilitation.

## Brain: traumatic brain injury and cognitive resilience

A number of the biological mechanisms of ketone bodies may be relevant to resilience of the brain under acute or chronic stress. Long-term non-obese ketogenic diets in mice have been shown to extend healthy lifespan most prominently by protecting cognitive function in aging [78, 79]. The neuroprotective potential of ketone bodies in diverse neurological diseases has been extensively reviewed [80–82], including specifically for traumatic brain injury (TBI) [82–85]. Modulation of acute maladaptive inflammation via NLRP3 and HCAR2, cytoprotection against oxidative stress, induction of resilience genes, and maintenance of cellular ATP via ketone oxidation might all play a role in resilience to TBI. These different mechanisms may operate in specific cell types (e.g., protection against neuronal death vs. inhibition of microglial activation) and at different times (e.g., reduced energetic stress during acute injury vs. reduced chronic maladaptive inflammation). In multiple, diverse rodent models of TBI, both prophylactic and post-injury ketogenic diet and exogenous ketone administration reduce cerebral edema, reduce apoptosis, improves cerebral metabolism, and improve long-term behavioral outcomes [85, 86].

Clinical studies of ketogenic diets or exogenous ketones related to the brain have mostly focused on resilience to or compensation for neurodegenerative disease of aging such as Alzheimer’s disease (AD). Small studies of medium chain triglycerides and ketogenic diets have demonstrated favorable effects on brain energetics, AD biomarkers, cerebral perfusion, and/or cognitive improvement [30, 31, 87]. A recent systematic review summarized 10 randomized controlled trials, which although highly heterogeneous in design and outcomes and clearly representing an early stage of research, show promise overall for AD [88]. A few small clinical studies specifically of ketones in military relevant settings such as TBI are now underway, largely at the safety/feasibility stage. Examples include an RCT of adolescents given ketone ester post-concussion at University of Alabama (NCT04079907), pilot studies of a ketogenic diet for patients with severe TBI in neurocritical care at University of Missouri (NCT03982602) and University of Copenhagen (NCT04308577), and a pilot study of MCTs for patients with severe TBI or subarachnoid hemorrhage in Switzerland (NCT02716532). Exogenous ketones are also being studied for their potential to mitigate hypoxia induced decrements in cognitive performance in pilots (NCT03659825).

## Muscle: anticatabolic, synthetic, and regenerative effects

An emerging potential application of ketogenic diet and ketone bodies is protecting against muscle loss in settings of starvation [13, 89], atrophy [90, 91], cachexia [91–93], or sarcopenia [78], as well as augmentation of muscle protein synthesis [94, 95] and damage-induced muscular regeneration [48]. The importance of this use case cannot be overstated for both the aging population, who rapidly lose muscle mass and function as a result of immobility [96], and the military population, who are at increased risk of muscle degradation or injury during training and combat [97, 98]. The “anti-catabolic” effect of ketone bodies is grounded in modulation of inflammation and cellular biochemistry and is consistent with the hypothesized evolutionary role of ketogenesis in sparing muscle catabolism during starvation by providing an alternative to gluconeogenesis for systemic energy supply [13, 89, 99]. Ketogenic diet used for weight loss in obese young military personnel causes loss of fat mass without significant changes in lean body mass or a military-specific set of physical performance tasks [24]. Both ketogenic diet and ketone body infusion reduce leucine oxidation, nitrogen excretion and alanine muscle efflux, and markers of protein catabolism, during starvation or an acute inflammatory insult [13, 89, 100–104]. Interestingly, ketone bodies were shown to have favorable effect on muscle protein turnover compared with glucose-induced hyper-insulinemia with LPS infusion [104], suggesting substitution of insulinogenic substrate for ketones may influence skeletal muscle anabolism in inflammatory environments. Altogether, exogenous ketones and ketogenic diet have been shown to reduce muscle wasting in a variety of cancer cachexia and inflammation-induced atrophy environments [91–93, 104–106].

In addition to reducing catabolism, there is some evidence that ketogenic diets or ketone bodies may promote protein synthesis in muscle via activation of the mTORC1 pathway. The interaction of ketogenic diet or ketone bodies with TOR is a complex topic that requires further detailed mechanistic investigation. In clinical studies, exogenous ketone bodies augment leucine-induced skeletal muscle protein synthesis [94, 95] via activation of the mTORC1 pathway [95]. Similarly, a lifespan study of ketogenic diet in mice showed attenuation of age-related muscle loss and preservation of muscle strength that were associated with markers of increased mTORC1 activation in skeletal muscle [78]. However, the effect of ketogenic diet or ketone bodies on TOR activity appears to be highly context- and/or tissue-dependent. The same mouse lifespan study found reduced TOR activity in the liver [78], and a second mouse lifespan study of ketogenic diet showed gene expression patterns consistent with reduced TOR activity in both liver and kidney [79]. In the diabetic kidney, ketone bodies are reported to inhibit aberrantly elevated mTORC1 signaling [107]. Finally, mTORC1 activity must be suppressed in the liver to permit hepatic ketogenesis during fasting [108]. The specific mechanisms by which ketone bodies interact with TOR pathway components remain to be elucidated.

These anticatabolic effects across dietary restricted inflammatory, sarcopenic, and cachexic environments, a protein synthetic role augmenting leucine-induced mTORC1 activation, and muscle cell proliferation and satellite cell regenerative effects in muscle hold clinical relevance across age-related diseases as well as muscle resilience, rehabilitation, and recovery. The molecular pathways thought to regulate these processes are multifaceted and include NLRP3, HCAR2, NFκB, HDACs, ubiquitination, mTORC1, MEK/ERK, and/or cellular NADPH pools [48, 58, 90, 91, 94, 95]. Early clinical trials at University of Bath (NCT03574987) and University of Rome (NCT04019431) are investigating body composition changes on low carbohydrate/ketogenic diets. Further clinical investigation of exogenous and endogenous ketones in the context of skeletal muscle anticatabolic, synthesis, and regeneration is needed and may prove fruitful for both aging and for operational muscle rehabilitation and resilience.

## Key gaps and limitations in current knowledge

The translation of ketone body biology from the bench to bedside and battlefield is still in the early stages. This translational science builds upon extensive and growing clinical experience, going back decades, with the use of ketogenic diets to treat epilepsy, childhood genetic disorders, and obesity and T2DM. Ketogenic diets are safe, accessible, and potently therapeutic but have inherent limitations as clinical interventions, including the complexity and feasibility of large-scale dietary changes, and the pleotropic effects that may or may not focus on key mechanisms for a specific condition. Recently, tools have emerged to provide ketone bodies more directly in the form of exogenous ketones: ketone salts, medium-chain triglycerides, and ketone esters. These too have limitations in palatability, cost, and the need for data on long-term use in both healthy young and aging populations. The pleotropic nature of ketogenic diet and exogenous ketones requires appropriate caution in designing clinical trials. Inhibiting acute inflammatory activation, increasing vasodilation, changes in blood chemistry, etc., may be helpful in some contexts but harmful in others (reviewed for the context of severe respiratory infection in [52]). Additional caution is warranted for patients or conditions where normal metabolic regulatory control is disrupted, to avoid harmful ketoacidosis.

The key scientific gap is to understand the particular molecular mechanisms that are most important for the application of ketone bodies in a specific clinical context (Table 1). This knowledge can then inform the choice and optimization of therapeutic interventions. For example, the body adapts in a comprehensive and coordinated manner to prolonged fasting or ketogenic diet, including increasing the efficiency of transport and utilization of fatty acids and ketone bodies for energy, especially in the brain. This adaptation may be helpful and would be encouraged in use cases that require oxidation of ketone bodies for energy, but counterproductive if the use case depends on drug-like direct protein binding activities and higher circulating levels of ketone bodies. It seems likely that adaptation is not required for many applications of exogenous ketosis; however, it is not clear if this applies in all situations. The efficacious dose, duration, timing, and pharmacokinetics of ketogenic therapy have not been clearly defined for most potential clinical interventions. For example, in the context of TBI would ketone bodies be most useful at improving pre-exposure resilience, mitigating injury during the exposure, or improving recovery after the exposure? Even how to define a “therapeutic level” of blood ketones is a unique question for a molecule whose primary route of clearance may be identical in some cases to its therapeutic action (oxidation to generate ATP). Ratios of the physiological ketone bodies, BHB and AcAc, can be manipulated by the choice of exogenous ketone compound, which could have implications for the energetic, signaling, and redox effects of each intervention. As ketone bodies both modulate and are dependent on whole-body metabolic function, there may be important interactions with diet and other factors such as age and metabolic health [109–111]. An understanding of key condition-specific mechanisms permits the selection or novel design of exogenous ketones to optimize each mechanism, perhaps in concert with adjuvant interventions to bolster other relevant downstream molecular pathways. In general, the tools and concepts already exist to proceed with informative proof of concept studies of ketogenic diet or exogenous ketones, but these questions will need to be worked out for individual clinical and field applications to optimize their therapeutic potential.

**Table 1:** Key research needs

Identify key specific mechanisms amongst pleiotropic ketone body effects in preclinical models	
Clinical proof-of-concept studies of ketogenic diet or exogenous ketones in specific conditions (e.g., TBI, muscle rehabilitation)	
Preclinical or clinical data on efficacious dose, duration, and kinetics of ketone bodies	
Interactions with diet, exercise, and other clinical variables	
Development of new candidate interventions with diverse target profiles	

## Summary

The pleotropic molecular effects of ketone bodies on mechanisms related to aging are an important emerging field with strong overlap with pathways implicated in military-relevant resilience and rehabilitation. Key molecular mechanisms include energetic support, attenuation of inflammation and oxidative stress, and effects on tissue fibrosis, stem cell function, and vascular function. While translation of the benefits of ketones in many of the hypothesized clinical and operational settings has yet to be validated, some areas have growing preclinical support. Areas that are particularly advanced include the use of ketone bodies in animal models of brain resilience and TBI, and muscle wasting/atrophy and damage across stressful environments. The tools and concepts exist to carry out early stage clinical investigations of ketogenic diet and exogenous ketones in these areas, and some clinical studies are already underway, but considerable work is needed to elucidate therapeutic effects. Preclinical models offer the best opportunity to rapidly study the mechanisms of action of ketone bodies across multiple applications, and speed the progress to well-designed clinical trials. Overall, ketogenic diet and exogenous ketones represent a rapidly translatable set of interventions with strong biological plausibility and some clinical proof of concept data to support investigation in both aging and military-relevant resilience and rehabilitation contexts particularly involving cognitive resilience, traumatic brain injury, and maintenance and recovery of muscle function through serious damage and/or illness.

## Abbreviations

AcAc: Acetoacetate
BHB: Beta-hydroxybutyrate (aka 3-hydroxybutyrate)
MCTs: Medium chain triglycerides
NLRP3: NOD-, LRR- and pyrin domain-containing protein 3
T2DM: Type 2 diabetes mellitus
TBI: Traumatic brain injury
